# Implementation and validation of DualSPHysics v5.4 for the simulation of four representative benchmarks of wave energy converters^☆^

**DOI:** 10.1016/j.mex.2026.104105

**Published:** 2026-08-26

**Authors:** Bonaventura Tagliafierro, Iván Martínez-Estévez, Salvatore Capasso, José M. Domínguez, Moncho Gómez-Gesteira, Moisés Brito, Daniel Clemente, Corrado Altomare, Giacomo Viccione, Jens Engström, Ryan G. Coe, Giorgio Bacelli, Georgios Fourtakas, Peter K. Stansby, B.D. Rogers, Malin Göteman, Alejandro J.C. Crespo

**Affiliations:** a Department of Electrical Engineering, Uppsala University, Uppsala, Sweden; b Environmental Physics Laboratory, CIM-UVIGO, Universidade de Vigo, Ourense, 32004, Spain; c Maritime Engineering Laboratory, Universitat Politècnica de Catalunya – BarcelonaTech, Barcelona, 08034, Spain; d Environmental and Maritime Hydraulics Laboratory (LIDAM), University of Salerno, Fisciano, 84084, Italy; e UNIDEMI, Department of Mechanical and Industrial Engineering, School of Science and Technology, Universidade NOVA de Lisboa, Lisboa, Portugal; f Department of Civil Engineering and Georresources, University of Porto, Rua Dr. Roberto Frias s/n, Porto, 4200-465, Portugal; g Kevin T. Crofton Department of Aerospace and Ocean Engineering Virginia Tech, Blacksburg, VA, 24061, USA; h Sandia National Laboratories, Albuquerque, NM, 87185, USA; i School of Engineering, The University of Manchester, Manchester, M13 9PL, UK

**Keywords:** Smoothed particle hydrodynamics, DualSPHysics, Wave energy converters, Numerical validation, Multibody dynamics, Mooring dynamics

## Abstract

This article describes the numerical framework implemented in DualSPHysics v5.4 for the high-fidelity simulation of wave energy converters (WECs), providing systematic re-validation, under a single current code version and with open-access input files, of four experimental benchmarks representative of the current state of the art in WEC technology. The framework integrates Smoothed Particle Hydrodynamics (SPH) for fluid dynamics, coupled with Project Chrono for rigid body and multibody dynamics, MoorDynPlus for mooring line dynamics, combined with a suite of wave generation tools. The four validated WEC configurations cover a broad range of device types, mooring arrangements, and power take-off systems. Quantitative agreement with experimental data is assessed using the normalized root mean squared error for three spatial resolutions for each case. All input files, executable configurations, and reference data series are made openly available under a CC BY 4.0 license.

• A multiphysics SPH-based simulation framework for wave energy converters, coupling fluid dynamics, rigid body motion, mooring dynamics, and power take-off behavior.
• Systematic validation against four experimental WEC benchmarks at three spatial resolutions.
• Simulation templates to support reproducibility with DualSPHysics.

## Graphical abstract

**Figure:**
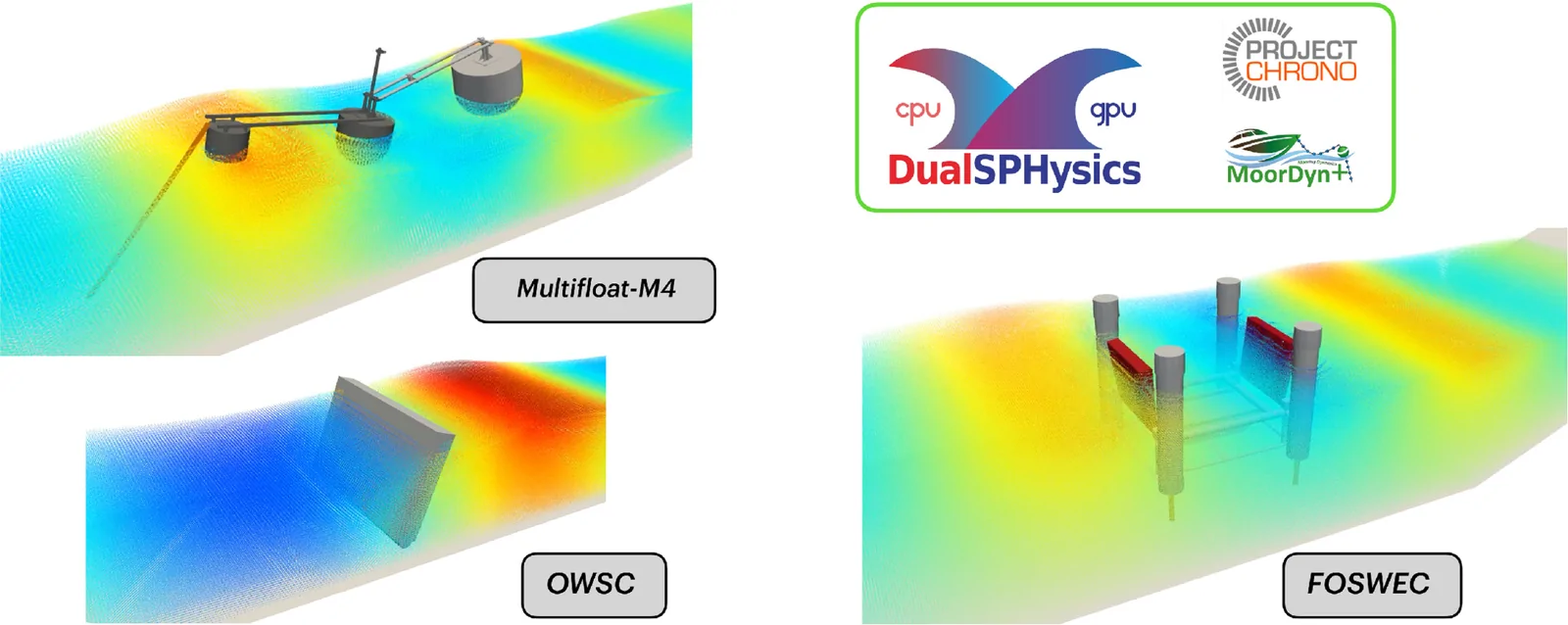

**Specifications table:**

**Table:** 

**Subject area**	Engineering
**More specific subject area**	*Computational fluid dynamics; offshore renewable energy; wave-structure interaction*
**Name of your method**	*High-fidelity SPH-based simulation framework for wave energy converters using DualSPHysics v5.4*
**Name and reference of original method**	*DualSPHysics: Domínguez* et al. [1]*, Computational Particle Mechanics,*https://doi.org/10.1016/j.cpc.2021.108250
**Resource availability**	*All simulation input files (XML metadata, STL geometry files, wavemaker signal files) and execution scripts are openly available at: Tagliafierro* et al. [2] https://doi.org/10.5281/zenodo.21793926*under a CC BY 4.0 license. The DualSPHysics v5.4 executable is available at*https://dual.sphysics.org/downloads/*under the GNU Lesser General Public License v3.*

## Background

Wave energy conversion remains one of the most promising yet technically challenging frontiers in offshore renewable energy. Despite a global wave power potential estimated at approximately 2 TW [3,4], WEC technology is yet to achieve the maturity needed for large-scale deployment [5]. Such a limited progress depends in part on the ability to model, with sufficient fidelity, the nonlinear interactions between waves and energy-harvesting structures, including fluid forces, PTO dynamics, and mooring response [6,7].

Numerical models for WECs range from low-fidelity potential flow solvers to fully nonlinear Navier–Stokes-based CFD tools [8,9]. While potential flow theory provides computational efficiency, it relies on assumptions, such as small body motion, small wave amplitudes, and negligible viscous effects, that WECs by design tend to violate, since significant body oscillations are needed to promote energy conversion [10]. High-fidelity CFD tools, including mesh-based solvers such as OpenFOAM [11], are well established for this purpose [12]; however, violent fluid–structure interaction can lead to mesh deformation and convergence difficulties under extreme conditions [13,14]. The Smoothed Particle Hydrodynamics (SPH) method [15] offers a natural alternative: as a mesh-free, Lagrangian particle method, it handles large free-surface deformations, moving boundaries, and complex multibody dynamics without the geometric constraints of mesh-based approaches [16,17]. Given their computational cost, high-fidelity CFD tools are not suited to iterative workflows typical of WEC control design and optimization, where reduced-order or potential-flow models remain the preferred choice. Their role in this context is instead to validate and/calibrate such reduced-order models against high-fidelity reference cases, or to resolve specific regimes, such as large-amplitude motion, violent free-surface events, or complex multibody/mooring interaction, where the assumptions underlying potential flow are known to break down. This aspect is treated in greater detail in the companion review paper, Tagliafierro et al. [18], where direct examples of control developments with SPH are reported.

## Method details

### The DualSPHysics’ implementation

For full 3-D simulations, Navier–Stokes equations describe fluid dynamics. In SPH, these equations are used to model the motion of discrete particles in a fully Lagrangian framework. By adopting a smoothing kernel function and a discrete summation over the particles included in the function’s support domain, the momentum equation results in the following expression:

(1) $$\frac{dv_{a}}{dt}=-\sum_{b}m_{b}\left(\frac{p_{a}+p_{b}}{\rho_{a}\rho_{b}}\right)\nabla_{a}W_{ab}+g+\Gamma_{a},$$

where subscript a indicates the measurement particle, b indicates the particles belonging to the kernel support, $W_{ab}$ is the kernel function, t is the time, v is the velocity, p is the pressure, and g is the gravitational acceleration vector. In Equation (1), the term Γ indicates treatment for viscous effects, the so-called laminar viscosity as formalized by [19]. It reads:

(2) $$\Gamma_{a}=\sum_{b}m_{b}\frac{4\upsilon_{0}r_{ab}\cdot \nabla_{a}W_{ab}}{\left(\rho_{a}+\rho_{b}\right)\left(r_{ab}^{2}\right)}u_{ab}.$$

In the SPH formalism, the discrete form of the continuity equation at a point a follows:

(3) $$\frac{d\rho_{a}}{dt}=\rho_{a}\sum_{b}\frac{m_{b}}{\rho_{b}}\;v_{ab}\cdot \nabla W_{ab}+D_{a}$$

where the second term on the right-hand side, $D_{a}$, represents a numerical density diffusion term introduced to reduce fluctuations in the density field, following the approach in Fourtakas et al. [20]. It is expressed as:

(4) $$D_{a}=2\delta h\;c_{s}\sum_{b}\left(\rho_{ba}^{T}-\rho_{ab}^{H}\right)\frac{r_{ab}\cdot \nabla_{a}W_{ab}}{r_{ab}^{2}}\frac{m_{b}}{\rho_{b}},$$

where $c_{s}$ is the speed of sound, δ is a real coefficient that controls the diffusive term, and superscripts T and H indicate the total and hydrostatic components of the density, respectively. The hydrostatic pressure is computed as $p_{ab}^{H}=\rho_{0}\;g\;z_{ab}$, where $z_{ab}$ is the position difference in *z* between particles a and b, and $\rho_{0}$ is the reference density.

DualSPHysics employs the so called WCSPH formulation that relates pressure to density through an equation of state, utilizing a conventional speed of sound ($c_{s}$), which is often lower than the physical one [21]:

(5) $$p=\frac{c_{s}^{2}\;\rho_{0}}{\gamma }\left[{\left(\frac{\rho }{\rho_{0}}\right)}^{\gamma }-1\right],$$

where γ is the polytropic constant (7 for water), and the numerical speed of sound that defines $\sqrt{\frac{\partial p}{\partial \rho }}$. To ensure density variations of less than 1% (resulting in low Mach numbers, Ma≈0.10), the numerical speed of sound is selected based on the domain’s characteristic length and time scales. This choice of numerical speed of sound permits larger time steps in the explicit time integration. However, there are certain applications, such as the generation and propagation of focused wave trains, which may benefit from higher values of the numerical speed of sound.

The implementation is completed via the time integrator scheme known as Symplectic (symplectic position Verlet) [22], which is an explicit and second-order accurate time scheme. In addition, a variable time step is enforced to satisfy the Courant–Friedrichs–Lewy (CFL) condition, where the force and the viscous diffusion terms follow the implementation in Monaghan and Kos [23]. Further details on the time integration scheme and the variable time step in DualSPHysics are given in Domínguez et al. [1].

### Boundary conditions

The boundary conditions (BCs) are implemented according to the formulation presented by English et al. [24,25] the so-called mDBCs (modified dynamic boundary conditions). This approach overcomes minor inconsistencies of previous implementations [26], such as large gaps between fluid and boundary particles that appear during the transition from non-wet to wet conditions. The particle layout for mDBC requires additional information to compute the solid–fluid interaction, using a boundary interface to locate the transition layer between the BCs and the fluid domain. This interface is used to enforce a mirroring technique using ghost nodes from the boundary particles into the fluid domain. The fluid properties are evaluated at these fictitious node positions; the SPH computation thus uses these values to consistently interpolate the solid particle quantities [27]. The use of mDBC ensures accurate pressure computations, as shown in English et al. [25]; Capasso et al. [28], and reduces the non-physical gap between boundary and fluid particles [29,30], observed in the former DBC (Dynamic BC) formulation [26].

### Rigid body dynamics

A key advantage of SPH models is their ability in simulating fluid-driven rigid objects. In DualSPHysics, this is achieved by calculating the forces exerted by fluid particles on a floating structure, defined as a subset of boundary particles, and using such forces to determine its motion. For each particle in the floating object, the net force is calculated by summing the contributions from surrounding fluid particles b. Then, assuming that a rigid body is discretized as a set of boundary particles, each boundary particle k experiences an acceleration (dv/dt), given by:

(6) $$\frac{dv_{k}}{dt}=\sum_{b}\frac{dv_{kb}}{dt}$$

Given the rigid body assumption, the motion of the object is determined by solving the fundamental equations of rigid body dynamics:

(7) $$M\frac{dV}{dt}=\sum_{k}m_{k}\frac{dv_{k}}{dt}$$

(8) $$I\frac{d\Omega }{dt}=\sum_{k}m_{k}\left(r_{k}-R_{0}\right)\frac{dv_{k}}{dt}$$

where M is the total mass of the object, I the moment of inertia matrix, V the linear velocity, Ω the angular velocity and $R_{0}$ the center of mass. Then, Equation (7) and Equation (8) are integrated in time to obtain the values of V and Ω at the next time step. Each boundary particle k belonging to the body move at a velocity $v_{k}$, given by:

(9) $$v_{k}=V+\Omega \times \left(r_{k}-R_{0}\right).$$

This technique has been further discussed by Monaghan et al. [31], where a detailed description is provided, proving that it ensures the conservation of linear and angular momentum.

### Coupling with Project Chrono

Martínez-Estévez et al. [32] presented a coupled multiphysics framework integrating Project Chrono and DualSPHysics, based on a former implementation introduced in Canelas et al. [33]. In this coupling, DualSPHysics handles fluid–solid interactions using SPH, while the multiphysics library Project Chrono [34] manages solid-solid interactions. In addition, Chrono performs collision detection with frictional contacts, applying forces to solid bodies. The accelerations of these bodies, determined by fluid forces from the SPH model, are then integrated by the multiphysics library, allowing for the simulation of complex, constrained multibody systems. A complete description of the coupling procedure is given in Martínez-Estévez et al. [32].

The numerical modeling of PTO system behavior in this work relies on the set of mechanical joints and actuators. Specifically, DualSPHysics v5.4 uses revolute joints from Chrono to handle articulated multibody systems by incorporating mechanical constraints into rigid body dynamics. In addition, it can introduce spring–damper actuators, which provide time-dependent forces based on relative displacement and velocity represented by a variable bilateral connection between two bodies. The direction of the applied force (${\hat{t}}_{sd}$) along such element is evaluated as:

(10) $${\hat{t}}_{sd}=\frac{r_{j}-r_{i}}{\left|r_{j}-r_{i}\right|},$$

where $r_{i}$ and $r_{j}$ are the positions of the joint points, *i* and *j*, respectively. The spring–damper produces a force:

(11) $$F_{c}=c_{c}v_{ij}\cdot {\hat{t}}_{sd}+k_{c}r_{ij}\cdot {\hat{t}}_{sd},$$

where $r_{ij}$ and $v_{ij}$ are the relative position and velocity between points *i* and *j*, respectively, and $k_{c}$ and $c_{c}$ are the spring stiffness and viscous damping coefficient.

Similarly, hinges or revolute joints constrain the motion to rotation about one axis, effectively eliminating translational degrees of freedom. A torque can be applied considering two rigid bodies with a certain θ, relative angle of rotation, and $\dot{\theta }$, angular velocity:

(12) $$M_{c}=k_{r}\;\theta +c_{r}\;\dot{\theta },$$

where $k_{r}$ and $c_{r}$ are the rotational stiffness and damping coefficients, respectively.

### Coupling with a mooring solver

DualSPHysics is coupled with MoorDynPlus [35], an open-source dynamic mooring line model implemented based on the main structure of MoorDyn [36]. MoorDynPlus allows solving catenary and taut-mooring lines.

This mooring solver discretizes lines as interconnected point masses with linear spring-dampers, providing axial elasticity and incorporating seabed contact forces. The model utilizes a lumped-mass approach, dividing the unstretched line length ($L_{0}$) into N equal segments, resulting in N+1 nodes. Each segment inherits properties from the overall line geometry, defined by parameters such as segment length (l = $L_{0}/N$), volume-equivalent area ($A=\pi d^{2}/4$, where d is the volume-equivalent diameter), and line material density ($\rho_{line}$). The net mass of each segment ($m_{i}$) is calculated as $Al(\rho_{line}-\rho_{w})$, where $\rho_{w}$ is the water density. The solver also incorporates the elasticity modulus and internal damping coefficient. This lumped-mass discretization allows for accurate modeling of the behavior of the mooring line based on its geometric and material properties. The complete coupling procedure for wave-induced motion of a moored structure can be found in Domínguez et al. [37] and Martínez-Estévez et al. [35].

### Wave generation with moving boundary

The DualSPHysics code has been developed to pursue applicability towards the simulation of free-surface flows including coastal protections and offshore structures. The code offers a robust suite of wave generation and absorption tools, enabling users to tailor simulations to specific requirements. This versatility is achieved through moving boundary particles, which accurately reproduce experimental wavemaker behaviors, including piston and flap types, and facilitate the generation of regular, random, solitary and focused waves.

For wave generation with moving boundary, DualSPHysics employs Biesel transfer functions [38] and second-order wavemaker theory, specifically drawing on Madsen [39], which provides a computationally efficient and accurate method for generating long second-order Stokes waves, for regular (monochromatic) and irregular (random) sea states. The implementation includes Hughes’ solution to suppress second harmonics [40], with limitations based on the Ursell number. For regular (monochromatic) waves, second-order correction is applied to remove superharmonics, which are the only higher-order components present, whereas for irregular (random) waves, second-order correction targets subharmonics, leading to the generation of infragravity waves.

The generation of focused wave groups is achieved using NewWave theory [41], which accurately describes the most probable shape of large waves in a given sea state, maximizing focused wave group energy at a specified location. Focused wave train is generated using an internal function in DualSPHysics [42,43], which implements a second order correction as developed by Schäffer [44].

To enable long time series simulations within short domains, DualSPHysics implements both passive and active wave absorption. Passive absorption utilizes a damping zone with a quadratic velocity reduction, detailed in Altomare et al. [45], to minimize wave reflection from domain boundaries. An active wave absorption system (AWAS), critical for eliminating re-reflection, is implemented for piston-type wavemakers. It uses a time-domain filtering technique, with free-surface elevation feedback at the wavemaker position. The wavemaker velocity is corrected to absorb reflected waves, based on linear long wave theory, as described in Schäffer [44] and Dean and Dalrymple [46]. This involves calculating the reflected free-surface elevation by comparing the measured and target incident elevations and then adjusting the wavemaker velocity accordingly. The detailed implementation of the method, including velocity correction and position adjustment, is documented in Altomare et al. [45].

### Numerical settings and error quantification

**Figure 1:**
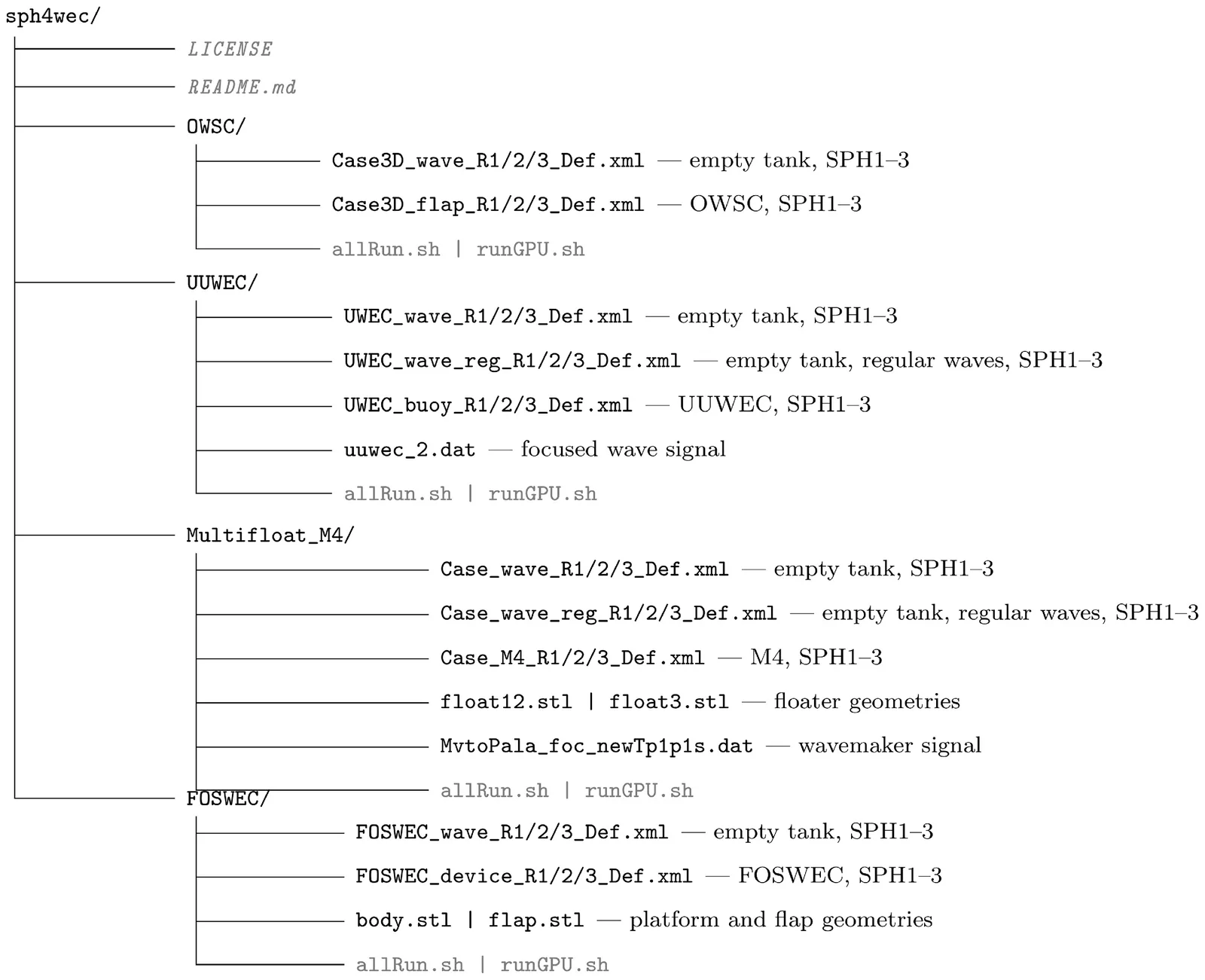
Schematic representation for the four wave tanks for the WEC validations.

During this validation segment, the devices selected as representative of the state-of-the-art in WECs include: i) an oscillating wave surge converter (OWSC) with a rotational power take-off (PTO) [47]; ii) a taut-line moored point absorber with a direct-drive, linear PTO (UUWEC) [48]; iii) a multibody attenuator with catenary-line mooring and a rotational PTO (Multi-float M4) [49]; and iv) a floating OWSC utilizing taut-line mooring and a rotational PTO (FOSWEC) [50]. Table 1 summarizes their key characteristics. Figure 1 illustrates the four models and their configurations.

**Table 1:** Relevant features of the four WEC designs.

	Name	Technology	Mooring	PTO type
1	IMFIA-OWSC	OWSC	None	Rotational
2	UUWEC	PA	Taut line	Linear
3	Multi-float M4	WA	Catenary line	Rotational
4	FOSWEC	OWSC	Taut lines	Rotational

These four devices have been re-validated under the same code version, based on the numerical configurations presented in their reference paper. The input files used by DualSPHysics (XML metadata) have been modified to accommodate the latest features for new implementations in version 5.4. Overall, the tests are reproduced using fresh water (initial density of $\rho_{0}$ = 998 $\text{kg}/m^{3}$). The speed of sound at the reference density $\rho_{0}$, $c_{0}$, is artificially set to $c_{0}=25\sqrt{gh_{SWL}}$, where $h_{SWL}$ indicates the initial water depth, ensuring less than 1% compressibility within the fluid domain. The smoothing length is defined as h = 2.08 Δp, where Δp denotes the initial particle spacing. For all the tests that follow, we have ensured the correct implementation of the mDBC theoretical requirements to the wave tank and the floater(s). The same DDT configuration, with a value of δ=0.10, is employed to dampen fluid pressure fluctuations that arise from the WCSPH implementation used in DualSPHysics. For each case, three different resolutions are used to perform a sensitivity analysis and ensure model consistency. In addition, the number of particles required per each simulation has been included along with wall-clock runtime, the real-time factor (RTF), and the particle-updates-per-second (PUPS), defined as the average number of active particles multiplied by the number of timesteps executed per second. All simulations were run on a workstation with an Intel 14900k (6.0 GHz), accelerated by an Nvidia GeForce RTX 5090 (32 GB of RAM), which sustained a throughput in the range of 130 million PUPS.

The numerical error between the computed and reference data was quantified using the normalized root mean squared error (ε). For a set x of N samples, the root mean squared error is defined as:

(13) $$\varepsilon \left(x\right)=\sqrt{\frac{1}{N}\sum_{i=1}^{N}{\left(x_{i}-x_{i}^{\text{ref}}\right)}^{2}},$$

where $x_{i}$ and $x_{i}^{\text{ref}}$ are the computed and reference values, respectively. Due to some time-offset between numerical and physical records, a single offset is calibrated for each simulation on the free-surface elevation recorded at the device location in the corresponding empty-tank test, by minimizing Eq. (13) over the offset itself. That offset is then imposed on the device response obtained with the same resolution, including body motions, relative rotations, and mooring tensions. Such lag is strongly dependent on the SPH resolution, arising from two separate mechanisms. First, SPH simulations may be sensitive to the size of the initial particle distance Δp, which is usually employed to generate the geometric setup on a lattice, cascading into approximated position of the solid boundaries. The second, directly results from the SPH operators, and specifically on the kernel support and number of neighbors (h/Δp) as established by Antuono et al. [51] for the δ-SPH scheme. Additionally, Colagrossi et al. [52], and more recently documented by Zago et al. [53], small values of h/Δp can give rise to excessive numerical dissipation, which shortens the propagating wavelength [53]. Through the linear dispersion relation, shorter wavelengths at fixed period correspond to reduced wave celerity, which can accumulate over the propagation distance from wavemaker to device, and manifests as the phase lag calibrated here.

The value of ε was then normalized using:

(14) $$\overset{‾}{\varepsilon }\left(x\right)=\frac{\varepsilon \left(x\right)}{\max\left(x^{\text{ref}}\right)-\min\left(x^{\text{ref}}\right)}.$$

This form allows for a clear comparison of errors across different magnitudes. For each validated device, these errors are reported as percentages in tables.

In addition to the point-wise error metrics defined above, wave reflection from the dissipative zone was quantified for each empty-tank test using the multi-gauge method of Zelt and Skjelbreia [54]. An array of five wave gauges was placed downstream of the device location, which assumes x_0_ at the device center of gravity. The remaining four gauges were spaced parametrically according to the wavelength λ of the tested regular wave, at x_1_ = x_0_ + 0.05λ, x_2_ = x_0_ + 0.125λ, x_3_ = x_0_ + 0.20λ, and x_4_ = x_0_ + 0.45λ, an unequal spacing pattern chosen to avoid the ill-conditioning that arises when gauge separations coincide with integer multiples of half the wavelength. The recorded elevation at each gauge is modelled as the superposition of an incident and a reflected wave, travelling in opposite directions — the method is applied here in the form adapted for periodic, single-frequency wave trains:

(15) $$\eta_{m}\;\left(t\right)\approx a_{m}\;cos\left(\omega t\right)+b_{m}\;sin(\omega t,\;$$

with *k* obtained from the linear dispersion relation $\omega^{2}=gk\;tanh\left(kd\right)$, and $a_{m}$, $b_{m}$ in Eq. (15) the in-phase and quadrature Fourier coefficients of the fitted harmonic at gauge *m*, so that $Z_{m}\;=\;a_{m}\;-\;ib_{m}$ is its complex amplitude (i is the imaginary unit). Writing the incident and reflected components in the same complex form, $Z_{m}\;=\;A_{I}\;e^{-ikx_{m}}\;+\;A_{R}\;e^{+ikx_{m}}$, the unknowns $A_{I}$ and $A_{R}$ are determined by least squares from the five $Z_{m}$. The incident and reflected wave heights follow as $H_{i}\;=\;2\left|A_{I}\right|$ and $H_{r}\;=\;2\left|A_{R}\right|$, and the reflection coefficient as $K_{R}\;=\;H_{r}/H_{i}$ . The free-surface elevation $\eta_{m}\left(t\right)$ was recorded at each gauge over a window of approximately ten wave periods once the signal reached a quasi-steady regime. For the focused-wave cases, this analysis is applied to a companion regular-wave test of the same tank rather than to the transient focused record.

### Simulation input files and repository structure

**Figure 2:**
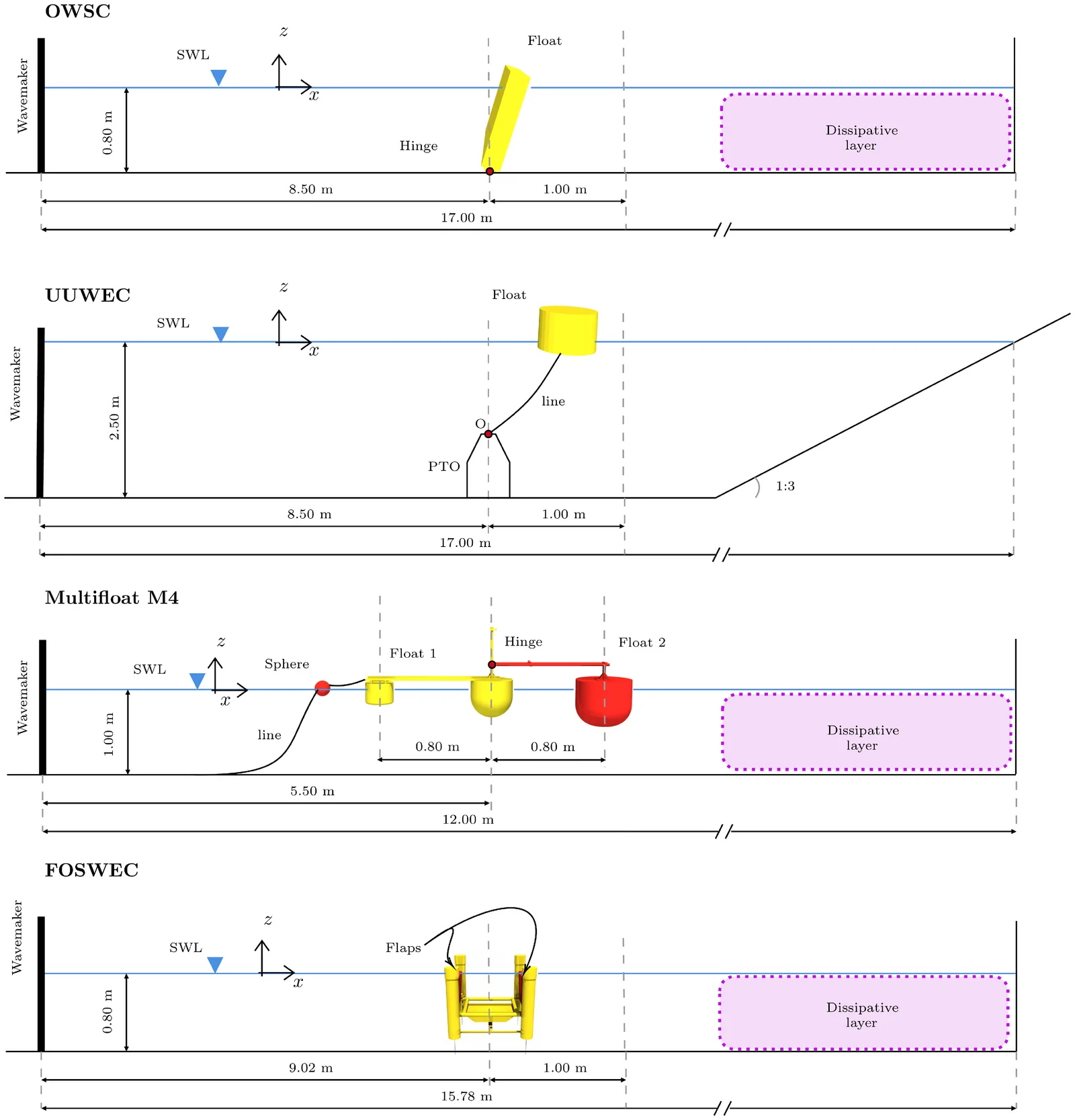
Repository structure for simulation input files Tagliafierro et al. [2].

The input files required to reproduce all simulations presented in this document are publicly available at Tagliafierro et al. [2] under a CC BY 4.0 license, also actively, including any future corrections or updates, on GitHub at https://github.com/btagliafierro/SPH4WEC. The repository is organized into four case-specific folders, one per WEC configuration, each containing the DualSPHysics XML metadata files, the geometry files in STL format where applicable, any external signal files required by the wavemaker, and shell scripts to run the cases. Figure 2 illustrates the directory layout, which includes a subfolder per each case, a license and a read-me file.

For each device, two or three sets of XML files are provided: one for the empty-tank wave propagation test and one for the full device simulation, both at three spatial resolutions (R1, R2, R3 corresponding to SPH1, SPH2, SPH3 throughout this paper). In addition, for the cases UUWEC and Multifloat-M4, equivalent regular wave cases are included for proper reflection analysis. Wave generation and propagation are first assessed independently before introducing the device, via local and global quality assessment. The XML files have been updated from their original configurations to be compatible with DualSPHysics v5.4 and include the mDBC boundary condition setup, the DDT density diffusion configuration, and the Chrono and/or MoorDynPlus coupling parameters.

The allRun.sh script runs all resolutions sequentially, while runGPU.sh executes a single user-specified case. The DualSPHysics v5.4 executable required to run these cases is available separately at https://dual.sphysics.org/downloads/ under the GNU Lesser General Public License v3.

## Method validation

### The IMFIA-OWSC

This first case refers to the validated setup presented by Brito et al. [47], in which experimental data is used to carry dynamic comparison of an OWSC under regular waves [55]. The physical testing took place in a wave flume at the Instituto de Mecánica de los Fluidos e Ingeniería Ambiental (IMFIA) in Uruguay. The floating part of the OWSC consists of an assembly of PVC tubes held together by a stainless-steel frame, which provides connections to a width-wise hinge at a height of 0.10 m above the wave tank bottom surface. The dimensions of the flap and its mass are given in Table 2. Note that its length indicates the distance from hinge to top of the flap, which according to the investigated water level, provides a freeboard of 0.03 m. The PTO system comprises a hydraulic cylinder and a closed hydraulic circuit connected to both the flap and the flume bed.

**Table 2:** Geometric and mechanical properties of the flap.

Element	Symbol	Value	Unit
Height	L	0.84	M
Beam	B	1.31	M
Width	W	0.17	m
Mass	m	72.3	kg
Moment of inertia	$I_{y}$	14.76	kg m^2^
Center of mass	$\left(x_{0},y_{0},z_{0}\right)$	(0, 0, 0.33)	m
Hinge position	(x,y,z)	(0, 0, 0.025)	m

The validation presented in Brito et al. [47] was conducted on four regular wave conditions that develop over a flat bottom tank with a water level of 0.825 m. The waves were represented by long-crested progressive waves reported as follows: Reg1 = ($H_{i}$ = 0.15 m, T = 2.00 s), Reg2 = (0.25 m, 2.00 s), Reg3 = (0.20 m, 3.00 s), and Reg4 = (0.25 m, 3.50 s). In Brito et al. [47], a particle spatial convergence analysis for wave propagation was performed on a 2-D setting to assess the choice of the numerical initial particle spacing (Δp) on the accuracy of the predicted free-surface elevations. The PTO system was also included in the modeling to account for the effects of friction and pressure forces within a hydraulic system that was driven by the flap pitch motion. The friction torque on the revolute joint between the flap and hinge bearings was modeled using an equivalent torque generated by a linear force proportional to the fluid pressure inside the hydraulic piston ([55] – see Chrono coupling section). For this work, the hydraulic PTO is represented as a Coulomb-damping link connecting the physical attachment points of the cylinder on the flap and on the floor, reproducing the actual piston geometry and line of action. The associated friction coefficient was calibrated against the Reg2 only and has not been re-validated against Reg1, Reg3, or Reg4 wave conditions reported in Brito et al. [47].

The Reg2 test condition, which specifies $H_{i}$ = 0.25 m, T = 2.00 s, depth = 0.825 m, λ = 4.25 m, has been simulated using a 3-D computational domain. For our validation, the numerical solutions obtained from three different resolutions are compared with the reference data; the main parameters employed to perform such sensitivity analysis for the OWSC are reported in Table 3. Specifically, this table reports three resolutions, SPH1, SPH2, and SPH3, respectively, which lead to two different levels of relative resolutions, regarding the wave propagation problem ($H_{i}/\Delta p$) and the solution of the wave–structure interaction. According to the flap geometry, the 4 layers that fit within the flap width for SPH1 can be considered barely sufficient for the correct implantation of the mDBC and DDT (on both sides). Instead, the same three levels of resolution lead to very conservative ratios for wave propagation (i.e., $H_{i}/\Delta p$ bigger than 6). Lastly, the first panel in Figure 1 provides a general overview of the wave tank configuration and the positioning of the device. For regular wave generation, a piston-type wavemaker was positioned upstream of the computational domain.

**Table 3:** Simulation parameters for the three resolutions utilized for the OWSC.

Parameter	SPH1	SPH2	SPH3
Δp [m]	0.04	0.03	0.02
$H_{i}$ [m]	0.25	0.25	0.25
$H_{i}/\Delta p$ [-]	8.333	12.5	25
W (flap width) [m]	0.17	0.17	0.17
W/Δp [-]	4.2	5.6	8.5
Particles [$\times 10^{6}$]	1.28	3.77	27.53
PUPS [$\times 10^{6}$]	114	128	130
Runtime [hour]	0.70	2.68	41.6
RTF [-]	60	241	3745

**Figure 3:**
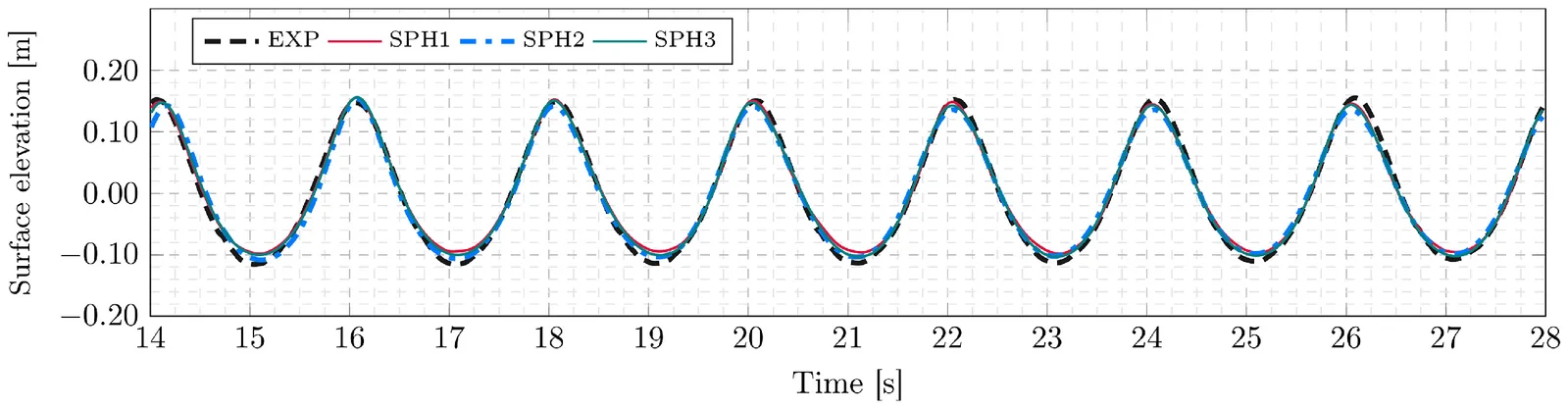
Free surface elevation comparison at the buoy location for the three resolutions.

Figure 3 presents a validation for regular wave generation and propagation for the three investigated resolutions. Wave free-surface elevation is sampled at two λs away from the piston, where the device would be later placed, providing a measure of the incident wave the device would be subject to. The three wave profiles suggest a good matching with the experimental series, demonstrating well matched profiles for the three resolutions, which is also confirmed by the error reported by the first row in Table 5. Table 4 quantifies this agreement for the same three simulations with only wave propagation. It shows that the wave period is almost perfectly recovered to within 0.04%, with lower incident wave heights, which however approach the target one with the SPH3. The reflection coefficient is 1.5% at all three resolutions, confirming quantitatively that the dissipative zone at the far end of the tank has been properly calibrated, as it absorbs the incident train effectively and causes acceptable reflection coefficient.

**Table 4:** Reflection analysis parameters and results for the OWSC wave tank.

Parameter	SPH1	SPH2	SPH3
Period error, ε(T) [%]	0.02	0.04	0.04
Incident wave height, $H_{i}$ [m]	0.239	0.231	0.245
Reflected wave height, $H_{r}$ [m]	0.004	0.003	0.004
Reflection coefficient, $K_{R}$ [%]	1.5	1.5	1.5

**Figure 4:**
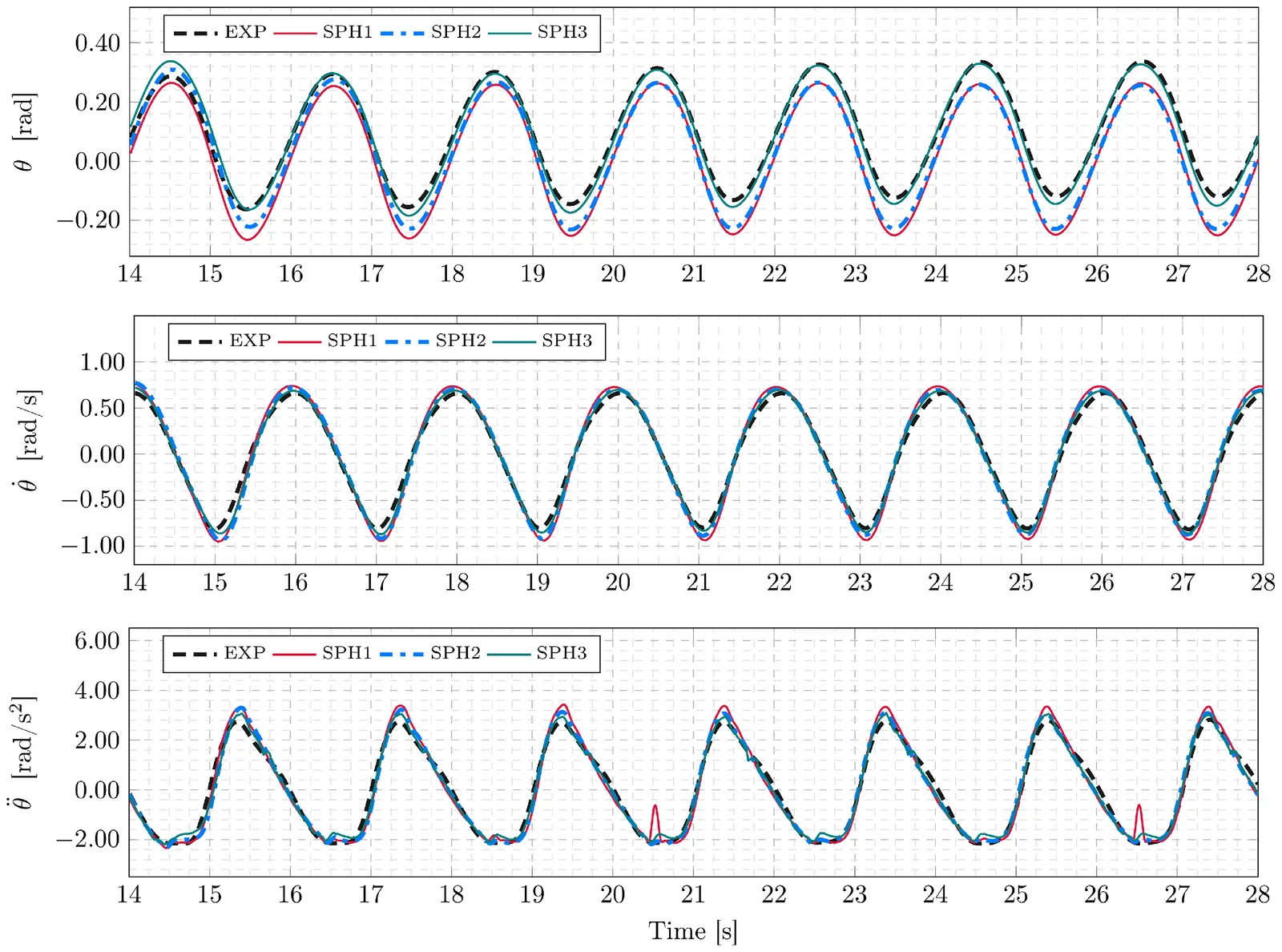
Comparison of the numerical and experimental time series of the angular position (θ), the angular velocity ($\ddot{\theta }$), and the angular acceleration ($\ddot{\theta }$) of the flap for the three numerical resolutions for the OWSC.

The three panels in Figure 4 compare the pitch position (θ), the angular velocity ($\dot{\theta }$), and the angular acceleration ($\ddot{\theta }$) of the flap with the experimental reference motion. The numerical model can accurately predict the dynamic behavior of the flap under cyclic excitation as confirmed by the error trends reported in Table 5. Pitch velocity and acceleration exhibit a clear converging pattern when the resolution decreases, whereas for the pitch angle, SPH1 and SPH2 show a large offset error generated by an underestimation of the mean equilibrium position (first panel in Figure 4), giving ε¯(θ) of about 15% (Table 5). At SPH3 the model however converges on the shifted equilibrium as well as on the oscillatory response, including the second-order pattern that forms when the flap recoils, and ε¯(θ) falls to 4.1%. The angular acceleration signal is smoothed with a third-order Savitzky–Golay filter prior to comparison; even so, $\ddot{\theta }$ retains some higher-frequency content at SPH3 than at the coarser resolutions. This is consistent with reduced numerical dissipation at finer particle spacing, which allows high-frequency pressure fluctuations to persist.

**Table 5:** Normalized root mean squared errors (ε) for the three spatial resolutions considered for the OWSC. Errors are reported as percentages.

Magnitude	SPH1	SPH2	SPH3
$\overset{‾}{\varepsilon }\left(\eta \right)$	3.6	3.9	3.0
$\overset{‾}{\varepsilon }\left(\theta \right)$	16.2	14.1	4.1
$\overset{‾}{\varepsilon }\left(\dot{\theta }\right)$	5.8	4.9	3.1
$\overset{‾}{\varepsilon }\left(\ddot{\theta }\right)$	6.8	5.2	4.4

### The Uppsala University WEC

The UUWEC has been validated using DualSPHysics, as presented Tagliafierro et al. [48], against an experimental setup presented in Göteman et al. [56]. As summarized in Table 6, its configuration comprised a cylindrical buoy of height 0.106 m, radius of 0.085 m and an initial draft of 0.030 m and connected to the main structure that mimics the functioning of a PTO, including end-stoppers, mechanical systems, and a mooring line. Two PTO configurations were considered, in which the harvesting system was not operational (internal damping 2.79 Ns/m), and when the harvesting function was activated (including extra friction resistance of 6.75 N). In the following, the validation for one wave condition and one PTO configuration is presented. Note that the wave flume, PTO numerical arrangement, and the mooring setup are given in Tagliafierro et al. [48].

**Table 6:** Geometric and mechanical properties for the buoy and translator for the UUWEC.

Element	Symbol	Value	Unit
Radius	$R_{b}$	0.085	m
Height	$h_{b}$	0.106	m
Draft	$d_{b}$	0.066	m
Mass	$m_{b}$	0.710	kg
Inertia	$I_{y}$	2.5 × 10^-3^	kg m^2^
Center of mass	$\left(x_{0},y_{0},z_{0}\right)$	(0, 0, -0.113)	m
Translator Mass	$m_{t}$	0.780	kg

This validation is performed using embedded focused wave trains that targeted the representation of an extreme event characterized by its crest main amplitude at $A_{cr}\left(x_{f},t_{f}\right)$, where $x_{f}$ and $t_{f}$ are the focusing position and focusing time, respectively, and wave period $T_{p}$=2.39 s, traveling over a 2.50-meter water depth. An overall schematic of the numerical wave tank is presented in the second panel of Figure 1. With these parameters for the regular wave train, combined with the 2.50-m water depth, it is possible to assert that the wave is traveling in intermediate water with a wavelength of $\lambda_{reg}=8.50$ m, and that it can be ranked as a second order Stokes’ wave. The phase between the regular background and the focused group is given as ϕ=π. Table 7 presents the main parameters in use for this validation.

**Table 7:** Simulation parameters for the three resolutions utilized for the UUWEC.

Parameter	SPH1	SPH2	SPH3
Δp [m]	0.0213	0.017	0.0142
$A_{cr}$ [m]	0.27	0.27	0.27
$A_{cr}/\Delta p$ [-]	12	16	19
$2\;\times R_{b}$ (Diameter)	0.17	0.17	0.17
$2\;\times R_{b}/\Delta p$ [-]	4	5	6
Particles [$\times 10^{6}$]	3.50	6.56	10.84
PUPS [$\times 10^{6}$]	116	120	122
Runtime [hour]	5.2	12.1	24.2
RTF [-]	381	890	1777

Table 8 reports the reflection analysis, performed on a companion regular-wave test of the same tank, where the wave height is set to 0.28 m and wave period 2.40 s. The wave period is recovered to within 0.1%, the incident wave height falls within 3% of the target across resolutions, and the reflection coefficient remains below 2%, confirming adequate absorption at the dissipative zone.

**Table 8:** Reflection analysis parameters and results for the UUWEC wave tank.

Parameter	SPH1	SPH2	SPH3
Period error, ε(T) [%]	0.05	0.06	0.07
Incident wave height, $H_{i}$ [m]	0.273	0.271	0.269
Reflected wave height, $H_{r}$ [m]	0.005	0.003	0.004
Reflection coefficient, $K_{R}$ [%]	1.8	0.8	1.4

**Figure 5:**
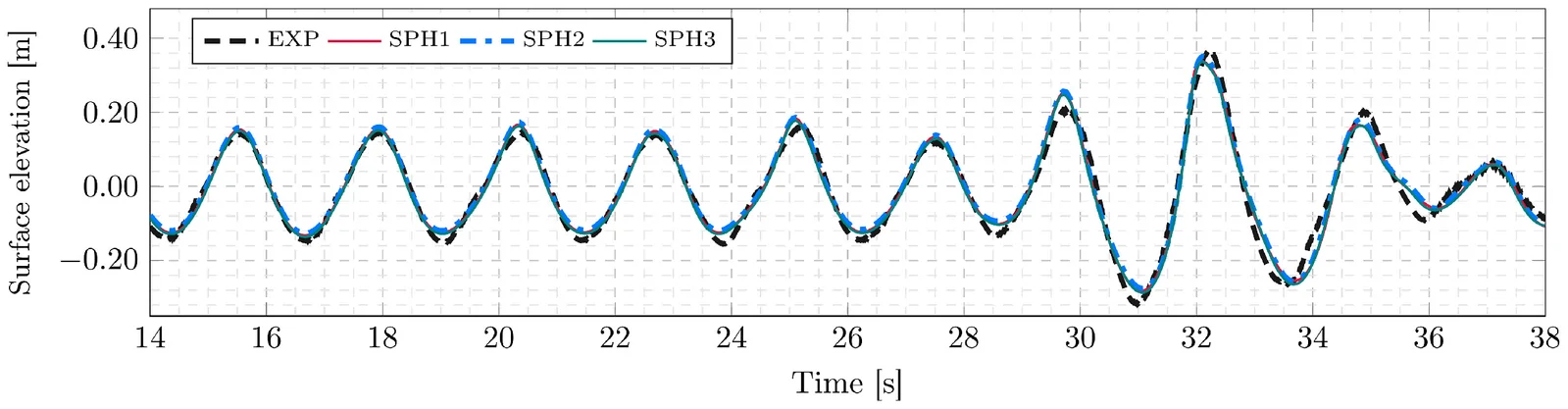
Free surface elevation comparison at the buoy's location for the three resolutions for the UUWEC.

Figure 5 compares the numerical results at three different resolutions with experimental data for the tested wave, measured at gauges positioned at the focusing location on an empty tank. By comparing the experimental measurements with the expected outcome, slight discrepancies can be observed around Time = 26 s, which may be caused by slight frequency differences in the input motion for both systems. In this case, the particle resolution is determined based on the float’s size to prepare the model for future applications and yields higher than standard values for wave propagation alone (see Table 9). As such, the evolution of the surface elevation closely follows the target trend, with minor deviations across the three resolutions. Major deviations between the numerical prediction and target expectations can be noted on the phase delay that occurs at the troughs of the focused group. Table 9 reports error metrics separately for the regular and focused parts of the record, split such that the regular train comprises t∈ (14, 26] s, and the focused group comprises t∈ (26, 38] s. ${\overset{¯}{\varepsilon }}_{reg}\left(\eta \right)$ is consistent with the regular-wave reflection test, showing an error about 3.1–3.4%, while ${\overset{¯}{\varepsilon }}_{foc}\left(\eta \right)$, at 2.0–2.4%, is lower despite the larger visual discrepancy noted at the group first troughs in Figure 5, since the metric is normalized by the larger range of the focused portion. In this validation, neither quantity shows a clear convergence trend across the three resolutions, which is consistent with the particle resolution here being well above the range typically required for wave propagation alone, so that the extra refinement refinements yield no additional benefit for the wave propagation validation.

**Table 9:** Normalized root mean squared errors ($\bar{\varepsilon }$) for the regular and focused segments of the record, for the three spatial resolutions considered for the UUWEC. Errors are reported as percentages.

Magnitude	SPH1	SPH2	SPH3
${\bar{\epsilon }}_{\text{reg}}\left(\eta \right)$	3.1	3.4	3.2
${\bar{\epsilon }}_{\text{foc}}\left(\eta \right)$	2.0	2.4	2.0
${\bar{\varepsilon }}_{\text{reg}}\left(\text{Surge}\right)$	7.3	7.0	7.0
${\bar{\varepsilon }}_{\text{foc}}\left(\text{Surge}\right)$	11.9	9.4	10.9
${\bar{\varepsilon }}_{\text{reg}}\left(\text{Heave}\right)$	4.6	3.9	5.6
${\bar{\varepsilon }}_{\text{foc}}\left(\text{Heave}\right)$	9.5	8.9	9.5
${\bar{\varepsilon }}_{\text{reg}}\left(\text{Tension}\right)$	5.8	5.5	5.1
${\bar{\varepsilon }}_{\text{foc}}\left(\text{Tension}\right)$	8.1	8.2	8.1

**Figure 6:**
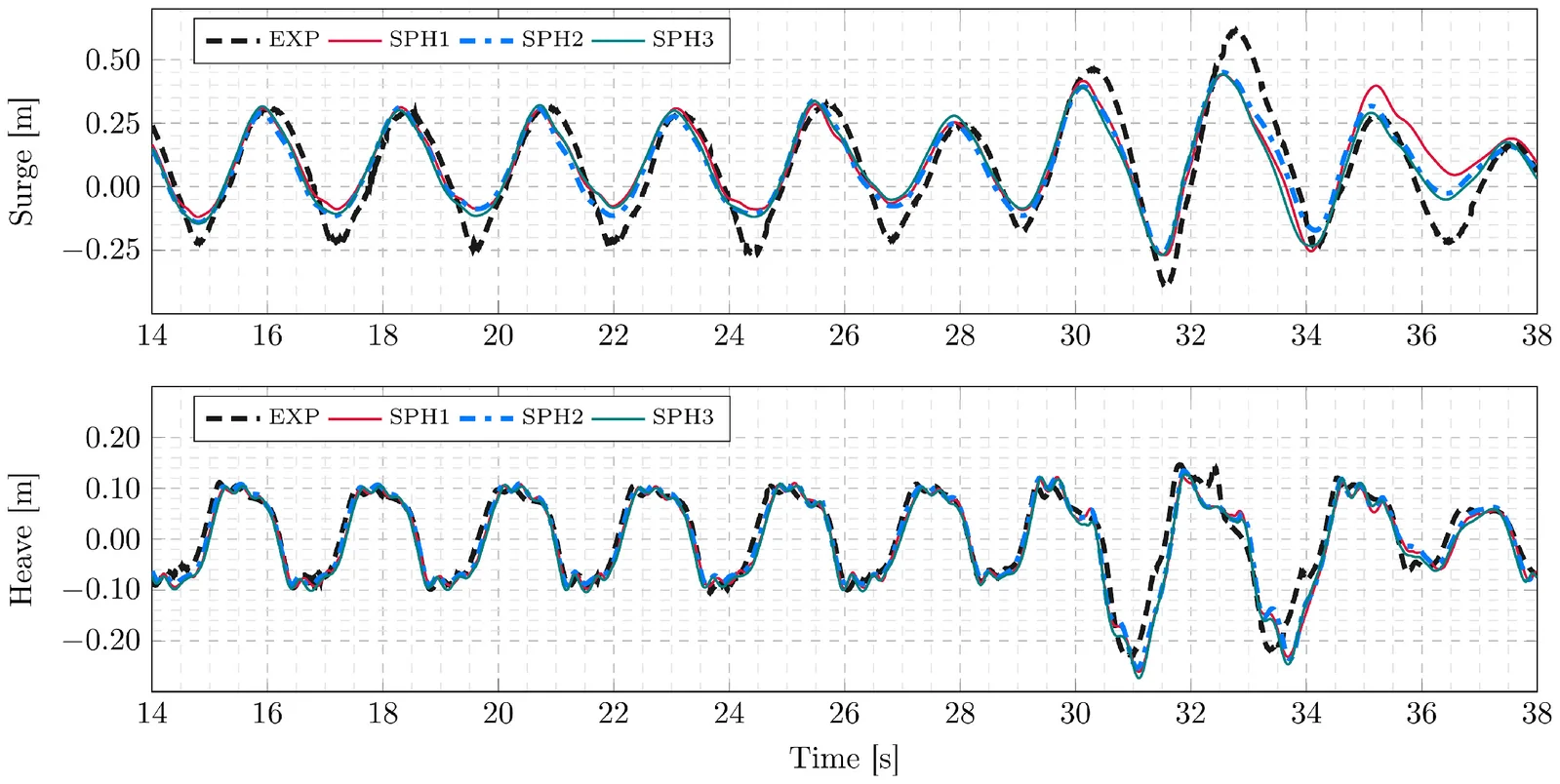
Experimental and numerical buoy position in surge (top panel) and heave (bottom panel) for the UUWEC.

Figure 6 shows the surge and heave buoy motion, which offer overall acceptable agreement in terms of frequency and amplitude response, with consistency across the three resolutions. Its first panel shows the surge motion validation, which presents a 20% underestimation in negative amplitude during the regular phase. Overall, the highest resolution (SPH3) does not resolve critical changes in the hydrodynamic response of the system compared to the medium one (SPH2). This consistent underestimation persists during the focused train, presenting a slight horizontal average drift. The heave evolution, in the second panel of Figure 6, matches almost perfectly with the experimental one across the preceding regular wave group, for all resolutions; minor inconsistency occurs at the troughs of the focused wave train, which mostly relates to the level of non-linearity of the PTO response.

This behavior is reflected in Table 9, which shows ${\overset{¯}{\varepsilon }}_{reg}$(Surge) essentially constant across resolutions, consistent with an underestimation that does not diminish with refinement, whereas ${\overset{¯}{\varepsilon }}_{foc}$(Surge) is larger and non-monotonic, mirroring the added horizontal drift noted during the focused train. By contrast, ${\overset{¯}{\varepsilon }}_{reg}$(Heave) remains below 6% at every resolution, and ${\overset{¯}{\varepsilon }}_{foc}$(Heave) is larger but consistent with the good regular-phase agreement and the more limited mismatch confined to the focused-train troughs described above. The large surge discrepancy in the focused-wave window does not resolve with refining resolution; however, this is not unique to the present framework. Sjökvist et al. [57] validated OpenFOAM and Ansys Fluent against the same experimental setup and wave condition, reporting average simulation errors of 13% and 21% respectively, despite very good agreement for wave propagation alone. Comparable response mismatches under focused-wave conditions have also been reported for another direct-drive linear PTO WEC device presented in Shahroozi et al. [58], and simulated with OpenFOAM [59] and DualSPHysics [60]. This consistency across independent solvers suggests that the discrepancy reflects more fundamental limitations, most plausibly in the buoy’s mechanical features, its PTO representation, or the mooring stiffness model.

**Figure 7:**
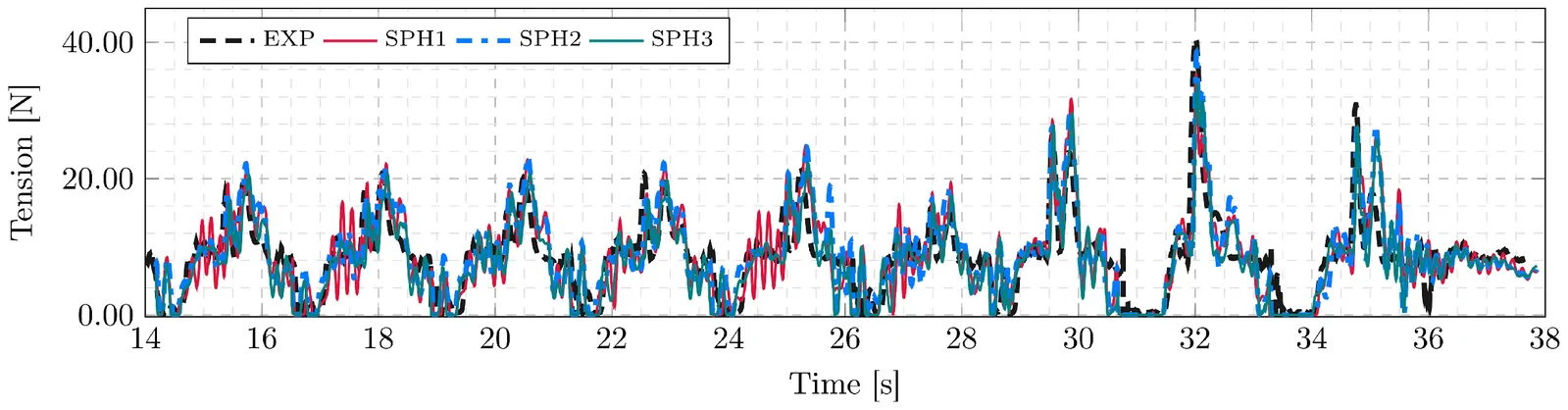
Experimental and numerical line force comparison for the UUWEC PTO.

Figure 7 presents the mooring line tension for the three simulated resolutions against experiments. Apart from SPH1, which shows some oscillations for the tension plateaus at end-stop engagement, the model captures all peaks quite accurately, as demonstrated by a small and constant error for all resolutions (Table 9). For SPH1, such differences are likely to be generated by sensitivity of the PTO system to resolution, as also detailed in Tagliafierro et al. [48]. For SPH2 and SPH3, the model correctly predicts the tension pattern under regular waves, showing a primary peak that corresponds to the compression of the end-stop and its rebound, which produces a secondary peak. Additionally, the slack events and their duration are also properly reproduced. Note that the tension time series have been shifted by 0.42 seconds to account for a phase shift between the motion and tension data for the experimental dataset [56,57].

### Multi-float M4

The device we study in this section, which is reminiscent of the one presented in [61], comprises three equally spaced floats, the size of which grows from bow to aft as reported in Table 10. The first version of this prototype underwent physical testing in the COAST (Coastal, Ocean and Sediment Transport) laboratory of the University of Plymouth (UK) [61]. This M4 develops around a revolute joint (a hinge), which connects the two main parts of this device. The bow-float and mid-float (Float 1) behave as a sole rigid body as there exists a structurally rigid connection between them, purposely designed to set this relative motion constraint; the stern float (Float 2) is connected by a rod to the central hinge on a vertical column on the mid-float (see Figure 1, third panel). This relative angular displacement was measured by an angular encoder. More details of the experimental tests can be found in [61].

**Table 10:** Geometric and mechanical properties of the structure’s components of the Multi-float M4.

Element	Symbol	Value	Unit
**Float 1**			
Diameter	$D_{1,bow}$	0.25	m
Diameter	$D_{1,middle}$	0.30	m
Mass	$m_{f1}$	10.68	kg
Center of mass	$\left(x_{1},y_{1},z_{1}\right)$	(5.345, 0, -0.059)	m
Inertia	${\left(I_{x},I_{y},I_{z}\right)}_{1}$	(0.16, 1.02, 0.96)	kg m^2^
**Float 2**			
Diameter	$D_{2,aft}$	0.40	m
Mass	$m_{f2}$	23.85	kg
Center of mass	$\left(x_{2},y_{2},z_{2}\right)$	(6.300, 0, -0.0983)	m
Inertia	${\left(I_{x},I_{y},I_{z}\right)}_{2}$	(0.41, 0.52, 0.53)	kg m^2^
**Buoy**			
Mass	$m_{s}$	0.087	kg
Diameter	$D_{s}$	0.100	m

An SPH numerical setup for the simulation of M4 was firstly presented by Carpintero Moreno et al. [49], where DualSPHysics was utilized to reproduce extreme wave conditions using a focused wave model [41]. Carpintero Moreno et al. [49] proposed a modeling and validation strategy employing three different wave sets, consisting of focused wave groups with peak spectral periods ($T_{p}$) of 1.00, 1.10, and 1.20 s, respectively, and defined a linear crest amplitude ($A_{cr}$) of 0.08 m (γ = 3.3). Their numerical configuration included two major modeling assumptions to downgrade the complexity of the case. First, the water depth was reduced to 0.60 m along the entire length of the tank, and secondly, the mooring connection comprised a single-point mooring line from Float 1 to the sea bottom.

In the present investigation, starting from the configuration previously examined, we have updated the geometrical layout and mooring configuration to match as close as possible the experimental one. As shown in the third panel of Figure 1, which sketches the numerical tank and localizes the M4, the simulated water depth corresponds to 1.00 m, whereas the anchoring system includes a single-point mooring line that intercepts a floating buoy 0.26 m before Float 1. The focused wave reproduced here, which complies with $T_{p}$=1.10 s and focal point ($x_{f}$) at the mid-float, is generated using a piston-type generator, moving according to a transfer function reconstructed from the properties reported in [61]. Table 11 reports the detailed resolution configurations for the validation that follows for the focused wave with $T_{p}$=1.10 s.

**Table 11:** Simulation parameters for the three resolutions utilized for the M4-MultiFloat.

Parameter	SPH1	SPH2	SPH3
Δp [m]	0.03	0.02	0.01
$H_{max}$ [m]	0.16	0.16	0.16
$H_{max}/\Delta p$ [-]	5.33	8.00	16.00
$D_{2,aft}$ [m]	0.40	0.40	0.40
$D_{2,aft}/\Delta p$ [-]	13.3	20.0	40.0
Particles [$\times 10^{6}$]	0.82	2.49	18.69
PUPS [× 10^6]	93	111	117
Runtime [hour]	0.38	1.44	20.6
RTF [-]	54	207	2973

Table 12 reports the reflection analysis, performed on a companion regular-wave test of the same tank targeting a wave height of 0.20 m, and wave period of 1.16 s. The incident wave height increases with resolution (0.110, 0.153, 0.170 m), and the reflection coefficient, while remaining low, grows slightly with refinement (0.3%, 1.5%, 2.6%), staying well within acceptable limits for the dissipative zone employed. This test indicates that SPH1 constitutes a very low resolution for the problem as it fails to propagate the wave consistently up to the buoy location (2.5 λs away from the piston. This extremely high dissipation is also mirrored by the very low reflection coefficient which for SPH1 is almost 0, proving that most of the energy is dissipated along the tank.

**Table 12:** Reflection analysis parameters and results for the M4-MultiFloat wave tank.

Parameter	SPH1	SPH2	SPH3
Period error, ε(T) [%]	0.18	0.11	0.45
Incident wave height, $H_{i}$ [m]	0.110	0.153	0.170
Reflected wave height, $H_{r}$ [m]	0.001	0.002	0.004
Reflection coefficient, $K_{R}$ [%]	0.3	1.5	2.6

**Figure 8:**
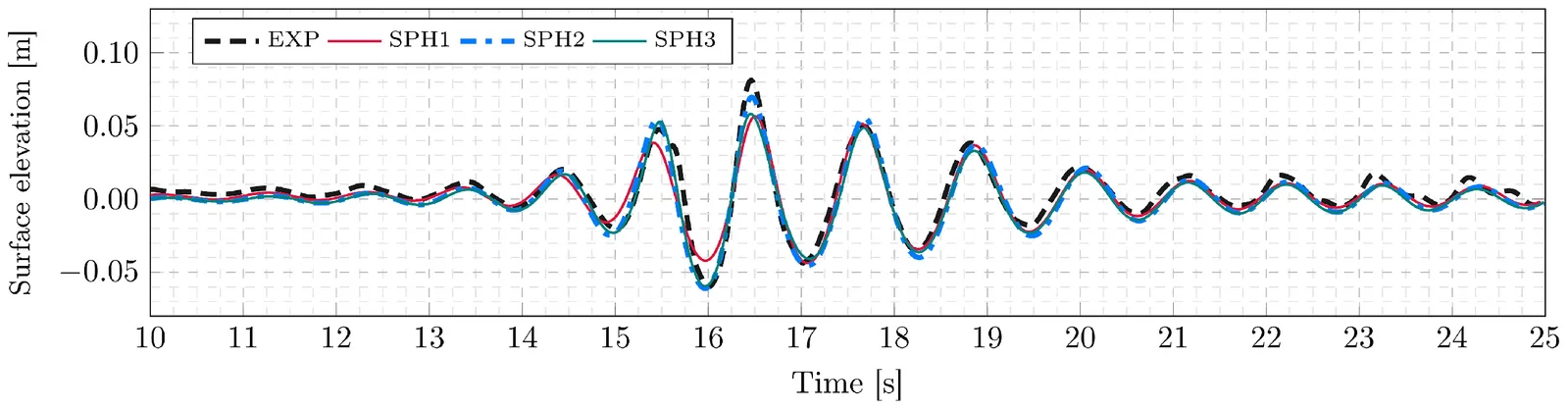
Free surface elevation comparison at the buoy’s location for the three resolutions.

Figure 8 presents wave generation and propagation using the wave tank defined in Figure 2, considering empty tank testing; this wave in generated using an imposed motion with signal reconstructions according to the properties illustrated in Carpintero Moreno et al. [49]. This figure compares the numerical time series of water-surface elevation with the measured data at the focusing location, which corresponds to the mid-float position. The proposed model in this work provides reasonable consistency in terms of frequency propagation, with more accurate wave profiles as the resolution increases. SPH1 produces smaller amplitudes at the first trough and main crest of the focused group, with a time delay measured of about 0.20 seconds for main crest, which is herein not visible because it has been accounted for according to the procedure mentioned in the method section. Again, such a big delay is likely caused by excessive numerical dissipation, which reduces the simulated wave lengths, and by pairing with a preserved wave period (see Table 12), implies reduced wave celerity. SPH2 and SPH3 deliver very similar wave profiles overall, reproducing, when compared to SPH1, better wave crests and troughs around the focused group (between 15.5 and 17.5 s).

**Figure 9:**
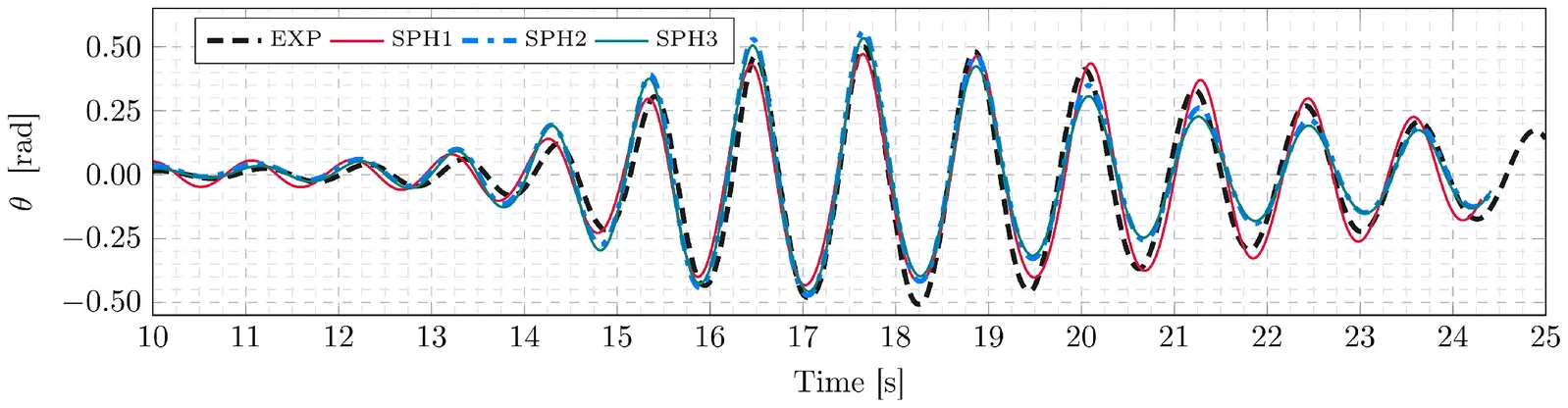
Relative pitch response for the Multifloat-M4 under a focused wave group.

Key to the modeling of this multi-float configuration is the use of the multi-physics library Project Chrono, and here specifically, its implementation in handling the relative rotational constraint between the two floats. Additionally, MoorDynPlus is used to connect, with a single-point mooring line, from Float 1 to a spherical buoy and from this buoy to sea bottom. In Figure 9, the relative angular displacement (θ) between the aft and bow floats is compared. Satisfactory agreement is achieved between the experimental angular response magnitude and the numerical simulation results for all particle resolutions, with SPH1 systematically deviating from the expected trend, producing a mismatch in frequency partly due to the shifted phases in the free surface (Figure 8). SPH2 and SPH3 are very well aligned in time with the reference response, matching quite well the motion amplitude. A slight phase shift is observed in the simulation during the last few seconds of the time series, likely due to the motion modes that dominate this part of the test. After 22 s, the input components have almost vanished and mainly the M4 is dissipating its kinetic energy through self-induced decay motion. Under these circumstances, higher dissipation is expected [62]. The error trend in Table 13 confirms the behavior noted before. Despite the small error values reported for SPH1, this resolution is not considered adequate for wave propagation, since it produces a measurable alteration of the incident wave field (Table 12). Most critically, a phase error can be detrimental to structures operating close to their natural frequency, such as WECs, where the response is highly sensitive to the wave frequency properties of the incident forcing.

**Table 13:** Normalized root mean squared errors (ε) for the three spatial resolutions considered for the M4. Errors are reported as percentages.

Magnitude	SPH1	SPH2	SPH3
$\overset{‾}{\varepsilon }\left(\eta \right)$	4.6	4.5	4.4
$\overset{‾}{\varepsilon }\left(\text{Pitch}\right)$	6.6	6.3	6.6

### The Floating Oscillating Surge Wave Energy Converter (FOSWEC)

The physical testing, designed by the Sandia National Laboratories (US), was carried out at the Directional Wave Basin at the Oregon State University (OSU) O.H. Hinsdale Wave Research Laboratory (HWRL). For validation purposes in [50], an energetic regular wave condition was selected and identified by Condition R5C in report [63]. The analyzed wave condition is taken from Test ID 194 [63], characterized as reported in Table 14. The water depth for this case is set to 1.36 m, hence the R5C wave train travels in intermediate water depths, meaning that the free surface profile is affected by the bathymetry. With respect to the wave height to be generated, we defined three levels of resolution, respectively, reported in Table 15. This last table, in addition, reports the reference dimension for FOSWEC, represented by an average flap width, $W_{flap}$, 0.05 m. The overall assembly of the numerical FOSWEC is visualized in the last panel of Figure 1.

**Table 14:** Selected wave conditions from the experimental setup R5C.

Parameter	Symbol	Quantity	Unit
Wave period	T	1.56	s
Wave height	$H_{i}$	0.136	m
Water depth	d	1.36	m
Wave length	λ	3.73	m

**Table 15:** Simulation parameters for the three resolutions utilized for the FOSWEC.

Parameter	SPH1	SPH2	SPH3
Δp [m]	0.03	0.02	0.015
$H_{i}$ [m]	0.136	0.136	0.136
$H_{i}/\Delta p$ [-]	4.5	6.8	9.0
$W_{flap}$ [m]	0.05	0.05	0.05
$W_{flap}/\Delta p$ [-]	<2	2.5	3.3
Particles [$\times 10^{6}$]	3.26	10.41	23.80
PUPS [$\times 10^{6}$]	117	131	130
Runtime [hour]	1.68	7.45	23.2
RTF [-]	201	893	2778

**Figure 10:**
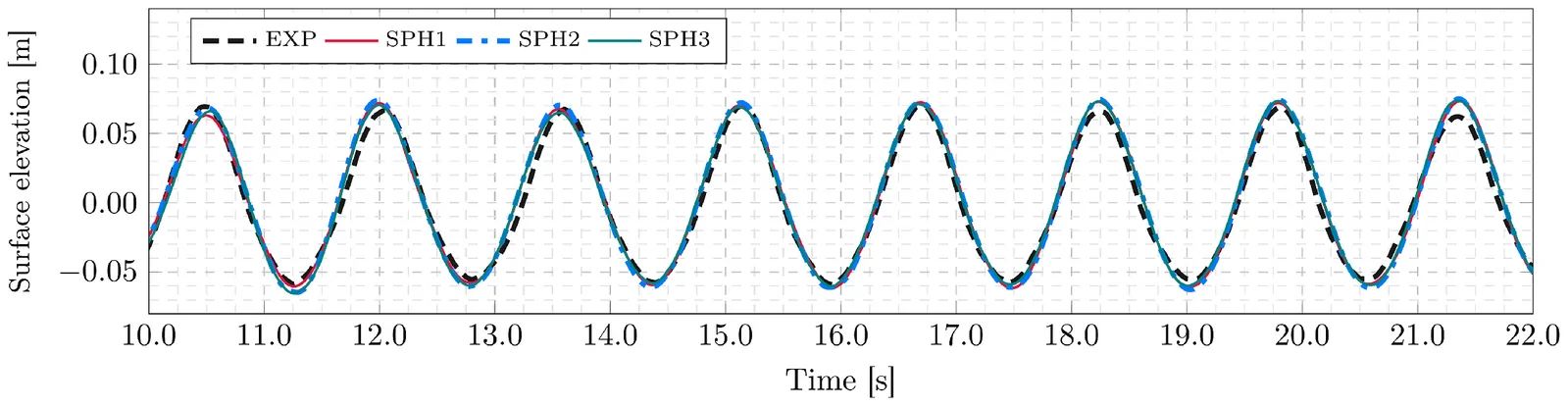
Free surface elevation comparison at the buoy’s location across the three resolutions for the FOSWEC wave tank.

Figure 10 presents a validation for wave generation and propagation for R5C for the three resolutions presented in Table 15. This figure compares the free-surface elevation at the buoy’s location in an empty tank test, using likewise information from the reference dataset. The model is shown to be quite consistent across all resolutions with very high accuracy in terms of reproduced period and wave amplitude. Table 16 confirms this quantitatively, showing that the wave period is recovered to within 0.2% at all resolutions, and the incident wave height is reproduced within 6% of the target $H_{i}\;=\;0.136\;m$, with SPH2 and SPH3 essentially indistinguishable. The reflection coefficient remains at or below 0.5% throughout, indicating that the dissipative zone absorbs the incident train effectively and that reflection does not contribute measurably to the residual ε¯(η) reported in Table 19. This agreement can be justified by the relatively high ratios in use, which start from almost five particles per wave height, and it is consistent with the near-constant range errors of about 5% obtained for all resolutions (Table 19).

**Table 16:** Reflection analysis parameters and results for the FOSWEC wave tank.

Parameter	SPH1	SPH2	SPH3
Period error, ε(T) [%]	0.05	0.12	0.15
Incident wave height, $H_{i}$ [m]	0.127	0.129	0.129
Reflected wave height, $H_{r}$ [m]	0.006	0.006	0.006
Reflection coefficient, $K_{R}$ [%]	0.5	0.5	0.4

The FOSWEC consists of a dual-flap device with a submerged central platform serving as the host for two flaps [64]. Four surface-piercing cylindrical columns, located at the four vertices of the platform, provide buoyancy, while the hull is anchored to the tank bottom via four taut cables arranged as tension legs. The two flaps, which are thin plates, are hinged to pivot around shafts mounted to the hull and can be controlled by independent motors to form the PTO. Figure 1 shows a 3-D view of the device assembly at its rest position; Table 17 presents the input parameters that define the mechanical and geometrical properties in simulation. Note that in this case, given the complexity of the platform geometry and the thickness of the flap, mDBC is only applied to the wavemaker and the walls of the tank, whereas the FOSWEC geometry is wholly solved using DBC.

**Table 17:** Input parameters used for the definition of FOSWEC platform.

Element	Symbol	Quantity	Unit
Mass	m	263	kg
Length	$L_{WEC}$	1.44	m
Beam	$W_{WEC}$	1.63	m
Draft	$D_{WEC}$	1.24	m
Center of mass	(x,y,z)	(0, 0, -0.7296)	m
Inertia	$\left(I_{x},I_{y},I_{z}\right)$	(43.46, 49.36, 7.75)	kg m^2^

The multi-body dynamics of FOSWEC is solved by Chrono. The two flaps are in relative motion to the hull via two revolute joints, which also provide spring–damper PTO behavior. Their parameters are defined with a spring term $k_{p}$ and a damping term $k_{d}$, which are used to define the time dependent quantity, $M_{a}$, that depends on the position and velocity of the flaps. Such parameters are derived from [63], considering a 30 s time window that spans t∈90−120 s in Test ID 194 [63]; the values of the control parameters for the defined batch are reported in Table 18. The negative stiffness values reported for both flaps reflect the reactive control strategy applied in the reference experimental campaign, in which the PTO actively supplies energy to the flap over part of each cycle. This window comprises 24 wave periods (T=1.56 s), of which 20 are regarded as well-developed and meaningful for this validation procedure. As mentioned, the platform is stabilized by four 15-cm tension legs, which absorb 540 N of buoyancy in still water, and are simulated using the MoorDynPlus library.

**Table 18:** Stiffness and damping coefficients for the PTO system.

Position	Parameter	Symbol	Quantity	Unit
Bow flap	Stiffness	$k_{p.b}$	-3.561	N·m/rad
Bow flap	Damping	$k_{d.b}$	1.762	N·m·s/rad
Aft flap	Stiffness	$k_{p.a}$	-2.800	N·m/rad
Aft flap	Damping	$k_{d.a}$	0.465	N·m·s/rad

**Figure 11:**
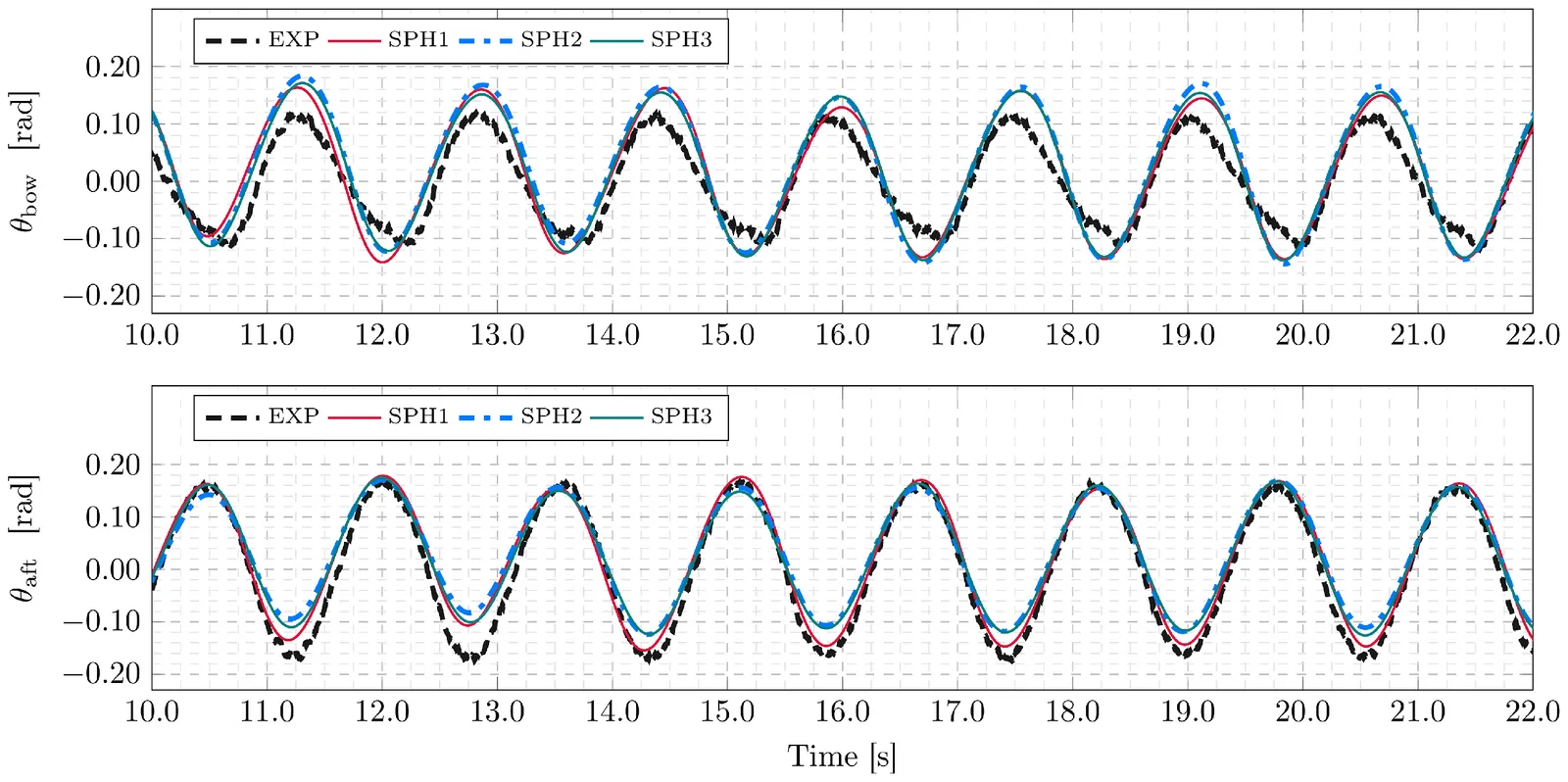
Experimental and numerical pitch comparison for the bow ($\theta_{\text{bow}}$) and aft flaps ($\theta_{\text{aft}}$). of the FOSWEC device.

Figure 11 shows the relative pitch motion of the numerical model, for three resolutions, for the two flaps against the experimental one, with the first panel referring to the bow flap ($\theta_{\text{bow}}$) and second to the aft flap ($\theta_{\text{aft}}$). The three resolutions produce broadly consistent motion responses that remain in phase with the experimental record throughout, with larger errors for the aft flap wave-wise motion and an overestimation of bow-flap amplitude of up to 16% (Table 19). These errors indicate a discrepancy that originates at the fluid–structure interface, consistent with the flap thickness being under-resolved at every tested resolution. The working requirement of approximately $W_{flap}/\Delta p$= 3 for consistent DBC treatment of a thin plate is not met here, yielding incorrect local fluid forces on the flap, further amplified by the reactive control system response.

**Table 19:** Normalized root mean squared errors (ε) for the three spatial resolutions considered for the FOSWEC. Errors are reported as percentages.

Magnitude	SPH1	SPH2	SPH3
$\overset{‾}{\varepsilon }\left(\eta \right)$	5.1	5.6	5.3
$\overset{‾}{\varepsilon }\left(\theta_{\text{aft}}\right)$	7.5	10.8	9.5
$\overset{‾}{\varepsilon }\left(\theta_{\text{bow}}\right)$	16.7	18.7	16.2
$\overset{‾}{\varepsilon }\left(\text{Tension}\right)$	29.1	26.3	16.7

**Figure 12:**
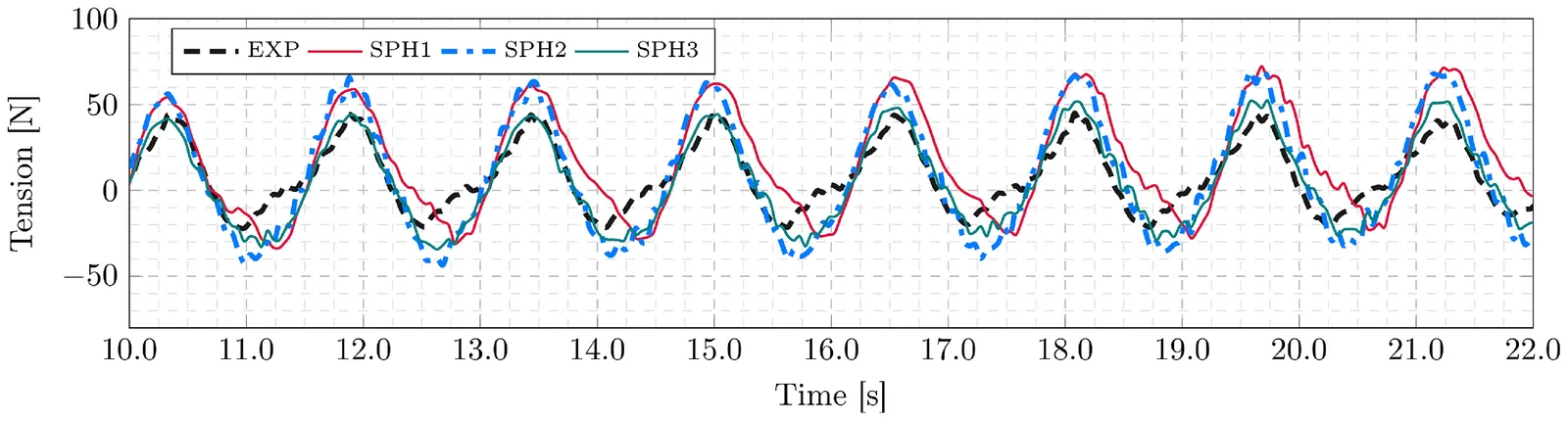
Experimental and numerical line tension comparison for the starboard tendon for the FOSWEC device.

Figure 12 compares the starboard cable net tension evolution for the three numerical resolutions: increased resolution yields more consistent responses, indicating a more accurate representation of FOSWEC overall behavior, with SPH1 deviating the most from the expected values, whereas SPH2 and SPH3 seem to produce closer tension values to the reference, with errors below 20% for the finest resolution (Table 19). When compared to the results in Tagliafierro et al. [50], better agreement is found here regarding the flap motion and tension evolution. Despite this improvement, the tension error remains the largest reported in this work and should be read in light of the flap under-resolution discussed above. The tension legs react to loads transmitted through the flaps and platform; the mooring response inherits the same fluid–force error. The FOSWEC is thus best regarded, among the four benchmarks presented here, as the case demanding the most caution, and as a demonstration of a reproducible setup comprising multi-body interaction and reactive control. However, further work is needed to provide proof of convergence and reliability to the presented setup.

## Limitations

The validations presented here have scoped four representative benchmarks under specific wave conditions, and the limitations that follow can be best read at two levels. First, there are limitations that have emerged directly from the present convergence studies, and more broadly, those that are intrinsic to the SPH method itself when applied to solve marine energy systems. A companion review [18] discusses the latter in greater details across the wider WEC literature. In the following, we focus on how those general limitations manifest concretely in the four cases validated in this work.

Resolution convergence is not uniform across the four presented benchmarks. For the IMFIA-OWSC and the UUWEC, the errors converge monotonically, confirming that these benchmarks are appropriately resolved at the finest tested resolution. For the Multi-float M4, the forced wave-driven response converges well, with a phase drift confined to the late-time, self-induced decay portion once the incident wave components have dissipated. The UUWEC surge motion additionally shows a persistent negative-amplitude underestimation (approximately 11%) that does not diminish with resolution, indicating that the cause lies in its geometrical and mechanical representation. The FOSWEC is the clearest case where resolution is the binding constraint, as the flap thickness remains under-resolved at every tested resolution. The resulting errors in flap motion and mooring tension show large discrepancies, due to the incorrect solution of the wave–fluid interaction, which is likely exacerbated by the reactive control system at the PTO level. At this point, it is also important to highlight that the limitation to the employed resolution comes from practical reasoning on execution times, here contained within a day or two on a personal workstation. This cost profile also delineates where high-fidelity SPH fits within a WEC design workflow. Indeed, SPH is intended as a complementary tool to test specific operating points, or explore possible non-linear regions, which are beyond the reach of engineering models.

Computational cost remains high relative to engineering-level or potential-flow solvers, which in practice restricts 3-D simulations to short physical durations, such as regular waves, or targeted design-wave events; this is reflected in the wider literature reviewed in [18] where only a small minority of studies performed 3-D simulations under irregular sea states. The weakly compressible formulation partially mitigates this cost by allowing an artificially reduced speed of sound while retaining acceptable accuracy, as done throughout this work, but this advantage narrows for full-scale or multi-scale simulations, where the particle resolution is bound by the smallest physical feature of the structure being simulated. The FOSWEC serves as a very compelling example for this.

Taken together, these observations point to two complementary directions for progress, consistent with the SPHERIC Grand Challenges on *adaptivity* and *coupling to other models* [65]. A first line of solutions sees SPH restricted to the solution of the near-field wave–structure interaction region while far-field propagation is delegated to computationally cheaper spectral or VOF-based solvers [66,67], configuring hybrid coupling strategies. Multi-resolution SPH, in which variable particle resolution within a single Lagrangian domain [68,69] could resolve small-scale features such as the FOSWEC flaps without incurring the cost of uniform fine resolution across the full domain, potentially enabling full sea-state simulations at engineering-relevant scales. Both directions are especially relevant to the FOSWEC-type case identified above as the current accuracy floor of this framework, as well as greatly speeding up the other cases, reducing the SPH geometrical size to one tenth of what is used now, with a greater saving in computational time.

## Supplementary material

The simulation input files associated with this work are described in the Method section are openly archived on Zenodo [2] at https://doi.org/10.5281/zenodo.21793926 under a CC BY 4.0 license. The actively maintained repository is available at https://github.com/btagliafierro/SPH4WEC.

## CRediT author statement

Bonaventura Tagliafierro: Conceptualization, Investigation, Methodology, Data curation, Visualization, Formal analysis, Writing – Original Draft. Ivan Martínez-Estevez: Writing – Original Draft, Writing – Review and Editing, Methodology, Software. Salvatore Capasso: Writing – Original Draft, Methodology, Visualization, Data curation, Formal analysis, Conceptualization, Writing – Review and Editing. José M. Domínguez: Software, Data curation, Validation. Moncho Gómez-Gesteira: Conceptualization, Writing – Review and Editing, Supervision. Moisés Brito: Model preparation, Writing – Review and Editing. Corrado Altomare: Writing – Review and Editing. Giacomo Viccione: Resources, Writing – Review and Editing. Jens Engström: Writing – Review and Editing. Ryan G. Coe: Data curation, Methodology, Writing – Review and Editing. Giorgio Bacelli: Writing – Review and Editing. Georgios Fourtakas: Writing – Review and Editing. Benedict D. Rogers: Data curation, Formal analysis, Writing – Review and Editing. Peter K. Stansby: Model preparation, Writing – Review and Editing. Malin Goteman: Data curation, Methodology, Investigation, Validation, Visualization, Writing – Review and Editing, Supervision. Alejandro J.C. Crespo: Conceptualization, Writing – Original Draft. Writing – Review and Editing, Model preparation, Visualization, Methodology, Data curation, Formal analysis, Supervision.

## Ethics statements

This work does not involve human or animal subjects; therefore, no ethical approval was required.

## Declaration of competing interest

The authors declare that they have no known competing financial interests or personal relationships that could have appeared to influence the work reported in this paper.

## Data Availability

Data is available along with the submission.
